# Prescription psychostimulants, atomoxetine and the risk of psychosis in adults with history of psychosis: a population-based cohort study

**DOI:** 10.1038/s41398-026-03998-4

**Published:** 2026-03-31

**Authors:** Patrick Bach, Johan Franck, Jonas Hällgren, Härje Widing, Mika Gissler, Jeanette Westman

**Affiliations:** 1 Department of Clinical Neuroscience, Karolinska Institutet, Stockholm, Sweden; 2 Department of Addictive Behavior and Addiction Medicine, Central Institute of Mental Health, University of Heidelberg, Medical Faculty Mannheim, Mannheim, Germany; 3 Academic Primary Care Centre, Region Stockholm, Stockholm, Sweden; 4 Department of Neurobiology, Care Sciences and Society, Karolinska Institutet, Stockholm, Sweden; 5 Department of Data and Analytics, Finnish Institute for Health and Welfare, Helsinki, Finland; 6 Department of Molecular Medicine and Surgery, Karolinska Institutet, Stockholm, Sweden; 7 Department of Health Care Sciences, Marie Cederschiöld University, Stockholm, Sweden

**Keywords:** Schizophrenia, Addiction, ADHD, Scientific community

## Abstract

Concerns exist that psychostimulants may increase the risk of psychosis, particularly in individuals with a history of psychosis. This study assessed whether psychosis risk increases after initiating psychostimulant or atomoxetine treatment in individuals with prior psychotic events. In this cohort study, we used Swedish population-based register data that contain data on all Swedish residents. We identified adults aged 18–64, who had a history of psychosis and initiated psychostimulant or atomoxetine treatment between 2008 and 2021. A within-individual design compared the rate of psychotic events in two 6-month periods before and after treatment initiation. Rate ratios (RR) with 95% confidence intervals (CI) were estimated. Sensitivity analyses were stratified by sex, age groups, and medication classes and also investigated substance-related psychosis as alternative outcome. We identified 3,770 individuals with a history of psychosis that received psychostimulant or atomoxetine prescriptions during the study period. Compared to the period before treatment initiation, the RR for psychotic events in the six months following treatment initiation was 0.95 (95% CI 0.84–1.08). Sensitivity analyses, considering only substance-induced psychotic events, or eight-week intervals and subgroup analyses by sex, age groups and separately for individuals receiving either (dex-)methyphenidate or (lis-)dexamphetamine or atomoxetine or (ar-)modafinil showed consistent results. Contrary to concerns, psychostimulant or atomoxetine treatment was not associated with an increased risk of psychotic events in adults with a history of psychosis. These findings may inform clinicians considering psychostimulant prescriptions in this population.

## Introduction

Prescription rates of psychostimulants, including (dex-)methylphenidate, (lis-)dexamphetamine (ar-)modafinil, and atomoxetine, are considerably high in Sweden [1], other Nordic countries [1] and worldwide [2] and pose an increasing concern regarding the risk of medication-induced psychosis and resulting physical and mental harm [3]. In Sweden, methylphenidate is the most frequently dispensed psychostimulant in adults, followed by lis-dexamphetamine and atomoxetine, whereas other amphetamine preparations and modafinil are used less common [1]. There are clinical concerns and experimental evidence that psychostimulants may worsen psychosis, especially in individuals with history of psychotic events [4–7]. The clinical challenge of managing the potential risk of psychostimulant treatment-emergent psychosis has become more pressing with increasing prescription rates of psychostimulants [2, 8–10].

A review of clinical trial data, commissioned by the US Food and Drug Administration, concluded that clinicians should be aware that psychosis could occur as adverse reaction during treatment with psychostimulants [11]. A 2018 Cochrane systematic review, which included 10 randomized trials (1 103 participants) and 17 non-randomized studies (76,237 participants) could not confirm or refute whether methylphenidate (MPH) increases the risk of psychotic symptoms [12]. Previous observational studies examining psychosis risk during psychostimulant treatment have reported mixed findings. A register-based study based on Swedish data (n = 23 898) reported no influence of MPH treatment on the incidence of psychotic events among individuals aged 12 to 30 years in the 12-week period after treatment initiation compared with the 12-week period preceding it (incidence rate ratio IRR = 0.95; 95% CI 0.69–1.30) [13]. A retrospective cohort study on Canadian health register data also investigated the risk of psychosis in individuals with psychotic disorders and comorbid attention-deficit hyperactivity disorder (ADHD). Again, authors reported a lower risk of hospital admissions for psychosis during the 12 months following initiation, when psychostimulants and antipsychotics were used in combination (HR = 0.36; 95% CI 0.24–0.54) [14]. In contrast, a population-based case-crossover study among n = 12 856 young individuals, who received a stimulant prescription, reported that initiation of psychostimulant treatment was associated with an increased risk of hospitalization for psychosis or mania in the subsequent 60 days (odds ratio OR = 1.86, 95% CI 1.39–2.56) [15]. In line with this, a case-control study (n = 1 374 cases, n = 2 748 controls) found that the odds of psychosis were increased for individuals aged 16–35 years with a past-month prescription for amphetamines (AMPH) (OR = 2.68, 95% CI = 1.90–3.77), while no significant increase in odds were observed for past-month prescription of MPH (OR = 0.91, 95% CI = 0.54–1.55) [16]. The risk was higher among individuals that received high versus low doses of AMPH. Further, a study utilizing data from two US large administrative claims databases (n = 221 846 adolescents and young adults up until the age of 25 years) reported that prescription of AMPH-type psychostimulants carried a higher risk of incidence psychosis compared to MPH (hazard ratio HR = 1.65; 95% CI 1.35 - 2.09), highlighting potential differences in psychosis risk between stimulant classes in non-psychotic populations [17]. This was supported by a pharmacovigilance study, investigating the association between psychotic symptoms and psychostimulants in patients aged 13–25 years. The study found that AMPH, compared to MPH use, was associated with an increased risk of reporting psychotic symptoms (relative odds ratio ROR = 1.61, 95% CI 1.26–2.06) [18]. Another retrospective study, using medical records–linkage system from a cohort of youth (age 6–18 years, n = 5,171) with diagnosed ADHD, found that exposure to AMPH-type psychostimulants (HR = 1.41, 95% CI 1.15–2.26) and atomoxetine HR = 2.01, 95% CI 1.38–2.92) was associated with increased risk of psychotic symptoms [19]. In the light of the mixed findings, a 2022 review of longitudinal observational studies concluded that observational studies do not support, nor refute an effect of prescription psychostimulants on psychosis risk [20].

Given these uncertainties, concerns have been raised that psychostimulants might increase the risk of psychosis, especially in individuals with history of psychosis. To date, the population of adults with history of psychosis has been understudied. Most observational studies of psychostimulants and psychosis risk have focused on children, adolescents and young adults with ADHD, and have typically excluded individuals with a prior history of psychotic disorders. The resulting evidence base therefore informs mainly on the risk of incident psychosis in largely non-psychotic ADHD populations, rather than the risk of relapse or exacerbation in adults with established psychotic disorders. This gap is clinically important, as clinicians must often weigh the potential benefits of psychostimulants or atomoxetine for attentional and functional symptoms against concerns about precipitating relapse in patients with a history of psychosis. Current clinical guidance regarding ADHD treatment in this group mainly rests on extrapolations from non-psychotic populations, small case series, and theoretical concerns, rather than on direct evidence. Some treatment guidelines even consider the use of stimulant medication, especially AMPH-like agents, to be contraindicated in patients with a history of psychosis [21, 22] with non-stimulant drugs, such as atomoxetine, being regarded as the recommended option [21, 22].

The presented work aimed to address the gap in knowledge concerning the risk of psychosis after initiation of psychostimulant or atomoxetine treatment among adults with a documented history of psychosis. To this end, we estimated the risk of hospitalizations for psychosis associated with initiation of psychostimulant or atomoxetine treatment among adults with a documented history of psychotic disorders by comparing periods before and after treatment initiation. The secondary objective was to explore whether the risk differed between specific medications, including MPH, AMPH-type psychostimulants, atomoxetine, and (ar-)modafinil.

## Materials and methods

### Study design and sample

**Fig. 1:**
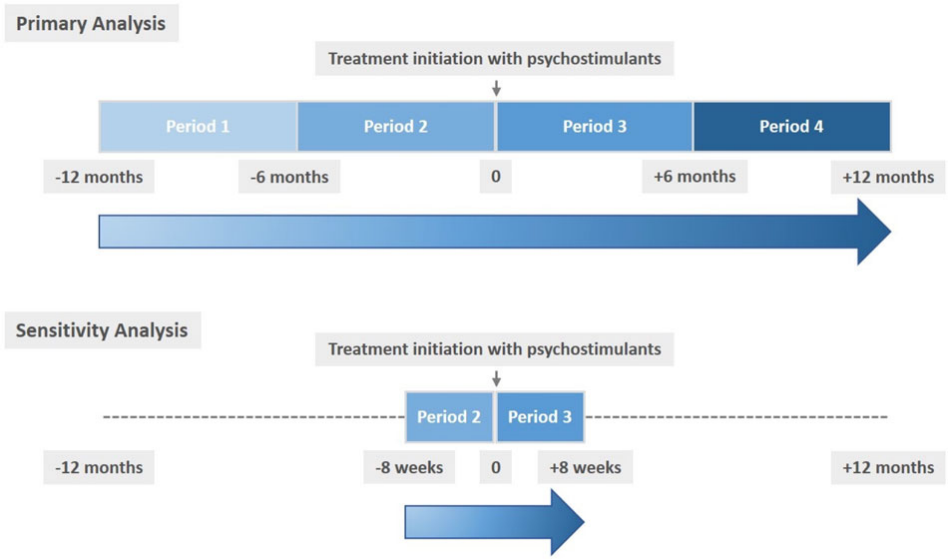
Illustration of the definition of study periods relative to treatment initiation that were compared with regards to the risk of psychotic events in the primary and sensitivity analysis.

This population-based cohort study in individuals with a history of psychosis that received a prescription for either psychostimulants or atomoxetine applied a within-individual study design to compare the rate of psychosis within the same individuals before and after initiation of treatment. To this end, we compared the rate of psychotic events during four 6-month observation periods that divided the year prior and after the index prescription in four equally long periods (P), i.e. P1 (month −12 to month −6 before treatment initiation), P2 (month −6 to the day before treatment initiation), P3 (day of treatment initiation to month +6 after treatment initiation), P4 (month +6 to month +12 after treatment initiation)(see Fig. 1).

From the Swedish registers, which include data on the whole population, we identified individuals that received a new prescription for psychostimulants or atomoxetine during the period between 2008 and 2021, were aged 18–64 and had a history of psychosis, which was defined as individuals with a registered diagnoses of a psychotic syndrome (ICD-10: F1X.5, F20, F22, F23, F25, F28, F29, F30.2, F31.2, F31.5, F32.3, F33.3, as recorded in the National Patient Register) during a period of −2 to −7 years prior to the start of the observational period.

New users were defined as individuals with a first registered dispensation of methylphenidate (Anatomical Therapeutic Chemical [ATC] code N06BA04), dexamphetamine (N06BA02), metamphetamine (N06BA03), modafinil (N06BA07), atomoxetine (N06BA09), dexmethylphenidate (N06BA11), lisdexamphetamine (N06BA12) or armodafinil (N06BA13) between Jan 1, 2008, and Dec 31, 2021, as identified from the Swedish Prescribed Drug Register. Clinically, stimulants are often considered more likely than atomoxetine to trigger psychotic symptoms, whereas the risk associated with modafinil/armodafinil is less well characterized despite their off-label use in some psychiatric populations. Including these agents allows for a more complete assessment and sub-group analyses for different medication classes.

New users included those who had initiated treatment with psychostimulants for the first time, who had not received any psychostimulant or atomoxetine for at least 1.5 years prior to the registered dispensation. This washout period aligns with evidence that adult ADHD medication treatment episodes typically last <1 year with high early discontinuation rates [1], and that stimulant-induced dopaminergic adaptations recover substantially over 12–18 months [23]. This definition thus identifies clinically meaningful new treatment episodes while reducing confounding from recent exposure, while maintaining representativeness to typical adult patients, many of whom have prior medication exposure from childhood or adolescence. Individuals were excluded if they had immigrated into Sweden after the start of P1, or if they had died or emigrated before the end of P4. In total of 50 individuals (1.3% of study population) died during the observational period and were excluded from the analyses. Major causes were suicide (n = 16) and accidents (n = 16).

The study was approved by the Swedish Ethical Review Authority at Karolinska Institutet (Stockholm, Sweden, decision id 2019-00516). Individual records were completely unidentifiable during the analysis. No informed consent was required since no registered person was contacted, and only anonymized data was used in the study. All methods were carried out in accordance with relevant guidelines and regulations governing the use of data from Swedish national registers.

### Data sources

The data for this cohort study were collected from registers with nationwide coverage. The Swedish Prescribed Drug Register [24], which contains data on all drugs that have been dispensed in Sweden since July 2005, was used to obtain data on dispensation dates, ATC codes (see above) and defined daily doses (DDDs). The Total Population Register [25] was used to obtain information on sex, age, and migration. The National Patient Register [26] provides information on contacts to inpatient and specialized outpatient services and the respective main diagnosis, which are coded according to the tenth revision of the International Statistical Classification of Diseases and Related Health Problems (ICD-10), with high coverage >96% for psychiatric main diagnosis that match well with patients records (positive predictive value of about 85–95%) [26]. It was used to identify hospitalizations due to psychotic events in the five years starting −1 year prior to the observational period, to group individuals into patients with and without history of psychosis, and to identify hospitalizations due to psychotic symptoms of an individual during the observational period. The National Cause of Death Register [27] was used to identify and exclude individuals that died during the observational study period.

### Outcomes

Psychotic events were the primary outcome and defined according to previous work [13] as any hospital visits (inpatient admission) with a main diagnosis of a psychotic event, according to the registered ICD-10 codes (F1x.5: substance-induced psychosis, F20: Schizophrenia, F22: Persistent delusional disorders, F23: Acute and transient psychotic disorders, F25: Schizoaffective disorders, F28: Other specified nonorganic psychotic disorders, F29: Unspecified nonorganic psychosis, F30.2: mania with psychotic symptoms, F31.2: bipolar affective disorder, current episode manic with psychotic symptoms, F31.5: bipolar affective disorder, current episode severe depression with psychotic symptoms, F32.3: severe depressive episode with psychotic symptoms, F33.3: recurrent depressive disorder, current episode severe with psychotic symptoms), as recorded in the National Patient Register [26]. In contrast to previous studies [13], we decided to not include organic psychotic syndromes (F06.0, F06.2) or general symptoms of perceptual disturbances (R44.0, R44.2, and R44.3) as we regard these codes as rather unspecific and/or likely related to external causes beyond pharmacological treatment (e.g. brain lesions, infections of the central nervous system).

As a secondary outcome, we used hospitalizations due to substance-induced psychosis (ICD-10 codes: F1x.5), as outcome which is closely linked to the effects of psychotropic substances.

In case the registered hospitalization periods overlapped, or discharge and admission were registered for the same day (e.g., due to transfer from one to another ward), such hospitalization events were not considered as new ones.

### Statistical analysis

To investigate the risk of psychosis after initiation of treatment, our primary analysis compared the rate of these events during the two 6-month periods on either side of treatment initiation (see Fig. 1), referred to as period 2 (P2) and period 3 (P3). Our secondary analyses compared the rate of psychotic events during the 6-month period immediately before psychostimulant initiation (P2) with that during the 6-month period starting at month six after treatment initiation (P4). For completeness, we also compared the rate of psychotic events during the 6-month period before treatment initiation (P2) with that during the 6-month period that started one calendar year before treatment initiation (P1).

Following previous work [13], the analyses used a conditional Poisson regression model to compare the rate of psychosis during the four different 6-month study periods (P1 to P4), with each patient as a separate stratum. Only individuals who had at least one psychotic event during the respective observational periods contributed data to the analysis. The results are presented as rate ratio (RRs) with 95% CIs. Hospitalization rates were calculated as the number of psychotic events per 10 000 person-weeks.

The influence of time-constant differences between individuals (e.g., sex, baseline disease severity) was adjusted for by conducting within-individual comparisons. To test the robustness of results from a 6-month period and assess potential transient immediate effects before and after treatment initiation, we performed sensitivity analyses setting the length of the observation period right before and after treatment initiation (i.e., P2 and P3) to be 8 weeks, instead of 6 months (Fig. 1).

We also conducted pre-planned subgroup analyses to investigate risk patterns across specific groups and medication classes. Specifically, we investigated the rate of psychotic events before and after initiation of psychostimulant treatment in both sexes (i.e., males and females), across different age groups (i.e., 18–34, 35–49, 50–64), in individuals with and without concurrent antipsychotic treatment during the two-year observational period (yes/no) and in different medication groups (dex-)methylphenidate, (lis-)dexamphetamine or metamphetamine, (ar-)modafinil, and atomoxetine.

We also repeated the primary analyses after excluding individuals with history of schizophrenia (F20.x), persistent delusional disorders (F22.x), schizoaffective disorders (F25.x), organic hallucinosis (F06.0), and organic delusional disorders (F06.2). This tested whether the main findings held in individuals with more episodic forms of psychosis, where clinicians may be more willing to prescribe psychostimulants, and removed individuals with aetiologies independent of medication effects.

Data management and statistical analyses were done with SAS version 9.4 and R software version 4.4.3.

## Results

We identified 3770 individuals with a history of psychosis who had started treatment with psychostimulants or atomoxetine between 2008 and 2021 and met the study inclusion criteria. Of those individuals, 2,340 (62.1%) were males. The mean age of the cohort was 35.3 years and on average individuals received 11.0 dispenses over the year after treatment initiation. A total of 753 (19.9%) had at least one psychotic event during the year prior or after treatment initiation (see Table 1).

**Table 1:** Study population characteristics (N = 3770), including all Swedish residents aged 18–64 years, with a psychostimulant prescription between 2008–2021 and history of psychosis.

	Individuals with history of psychosis (N = 3 770)
**Age, mean (SD)**	35.25 (10.1)
**Sex, n (%)**	
Male	2340 (62.1)
Female	1430 (37.9)
**Psychostimulant class prescribed at treatment initiation, n (%)**	
dexamfetamine or lisdexamfetamine or metamfetamine	412 (10.9)
atomoxetine	780 (20.7)
methylphenidate or dexmethylphenidate	2378 (63.1)
modafinil or armodafinil	200 (5.3)
**DDDs first year, mean (SD)**	511.9 (573.5)
**Number of dispenses in the first year after initiation of treatment, mean (SD)**	11.0 (9.2)
**Number of hospital admissions during two-year study period due to psychosis, n (%)**	
0	3017 (80.0)
1–2	507 (13.4)
3–5	177 (4.7)
≥6	69 (1.8)
**Main diagnosis of hospital admissions, n (%)**	
At least one psychosis due to substance use (ICD-10 codes: F1x.5)	212 (5.5)
Other psychosis diagnoses (ICD-10 codes: F20, F22, F23, F25, F28, F29, F30.2, F31.2, F31.5, F32.3, F33.3)	700 (17.8)
**Psychopharmaceutical classes dispensed +/- 3 days from initiation of treatment with psychostimulants, n (%)**	
Opioids (ATC code N02A)	100 (2.7)
Other Analgesics and Antipyretics (ATC code N02B)	246 (6.5)
Antiepileptics (ATC code N03A)	289 (7.7)
Antipsychotics (ATC code N05A)	844 (22.4)
Anxiolytics (ATC code N05B)	452 (12)
Hypnotics and Sedatives (ATC code N05C)	828 (22)
Antidepressants (ATC code N06A)	643 (17.1)
Drugs Used in Addictive Disorders (ATC code N07B)	129 (3.4)
**Dispensing of antipsychotics (ATC code N05A) during the different observation periods, n (%)**	
P1	1827 (48.5)
P2	1898 (50.3)
P3	1982 (52.6)
P4	1908 (50.6)

ICD-10 = International Statistical Classification of Diseases and Related Health Problems 10th Revision, P1: the 6-month period starting 1 calendar year before treatment initiation. P2: the 6-month period before treatment initiation. P3: the 6-month period after treatment initiation. P4: the 6-month period starting 6 months after treatment initiation.

**Fig. 2:**
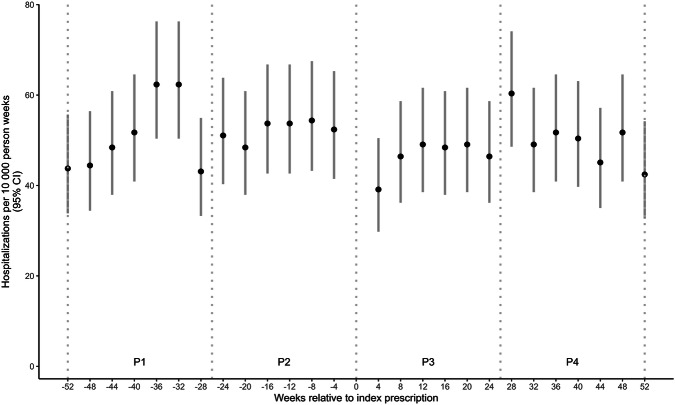
Rate of psychotic events per 10,000 person-weeks, during the 52 weeks before and after psychostimulant treatment initiation among individuals with a history of a psychosis (N = 3770) (CI = Confidence Interval, P = Period).

The overall rate of psychotic events was 50.07 events per 10,000 person-weeks (95% CI 47.88–52.33). The rate of hospitalizations attributed to psychotic events during the 52 weeks before and after initiation of psychostimulant treatment are shown in Fig. 2.

**Fig. 3:**
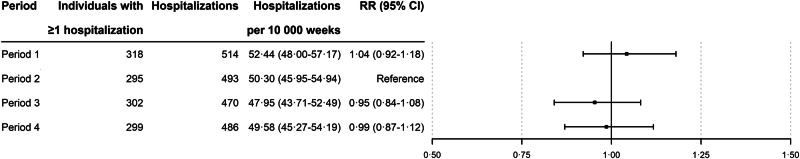
Depiction of the rate ratio of psychotic events for individuals with history of psychosis (N = 3770) during different periods before (period 1) and after initiation (period 3 and 4) of psychostimulant treatment (reference: period 2). (Period 1: the 6-month period starting one calendar year before treatment initiation. Period 2: the 6-month period before treatment initiation. Period 3: the 6-month period after treatment initiation. Period 4: the 6-month period staring 6 months after treatment initiation; CI = Confidence Interval, RR=Rate Ratio).

The risk of hospitalization for psychosis was not higher in the six months following initiation of a psychostimulant or atomoxetine than in the six months before initiation. Specifically, there were 493 psychotic events in the period before treatment initiation (P2) compared to 470 events in the period after treatment initiation (P3), corresponding to a rate ratio of 0.95 (95% CI 0.84–1.08; see Fig. 3).

For the medium-term follow-up (6–12 months after initiation, P4), we similarly found no increase in psychotic events (486 events) compared to the period before initiation (P2 vs P4: RR 0.99, 95% CI 0.87–1.12; see Fig. 3). Additionally, event rates in the year before treatment initiation (P1: 514 events) were stable compared to the 6 months prior to initiation (P1 vs P2: RR 1.04, 95% CI 0.92–1.18; see Fig. 3), indicating no marked secular trend or anticipatory rise in psychosis rates prior to drug initiation.

When we examined a shorter observation window (8 weeks before and after initiation), results were consistent, with a point estimate even suggesting a 15% reduction in psychotic events shortly after treatment start, though this did not reach statistical significance (RR 0.85, 95% CI 0.68–1.05; see Supplementary Figure S1).

**Fig. 4:**
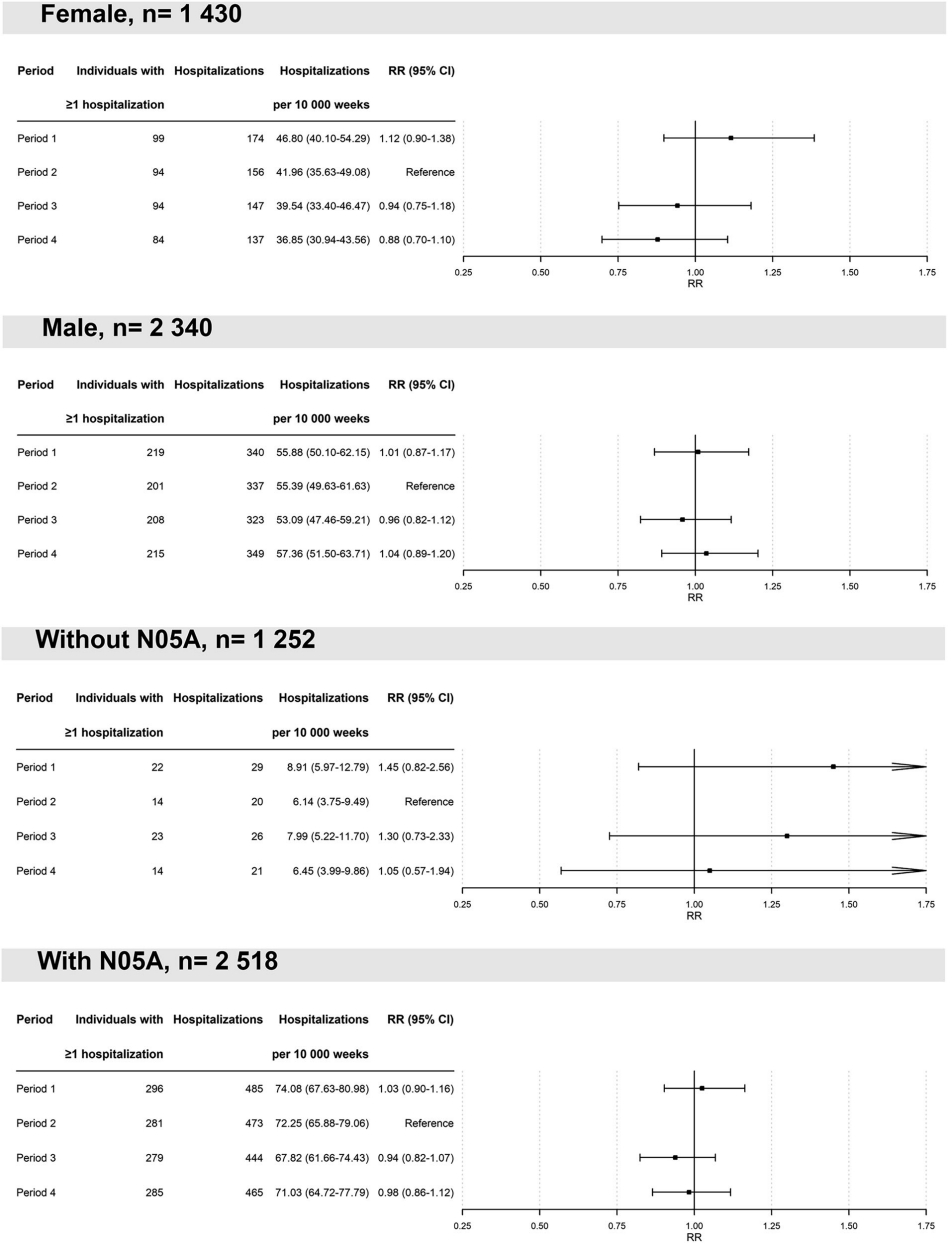
Depiction of the rate ratio of psychotic events before (period 1) and after initiation (period 3 and 4) of psychostimulant treatment (reference: period 2) for different subgroups, including females (N = 1430) and males (N = 2518) and also individuals with (N = 2518) and without (N = 1252) prescription of antipsychotics (ATC code: N05A) during the observational period. (Period 1: the 6-month period starting one calendar year before treatment initiation. Period 2: the 6-month period before treatment initiation. Period 3: the 6-month period after treatment initiation. Period 4: the 6-month period staring 6 months after treatment initiation; CI = Confidence Interval, RR=Rate Ratio).

Subgroup analyses in both sexes revealed no increased psychosis risk in either males (337 events pre-initiation vs. 323 post-initiation; RR 0.96, 95% CI 0.82–1.12) or females (156 vs. 147 events; RR 0.94, 95% CI 0.75–1.18; see Fig. 4).

Results regarding concurrent antipsychotic treatment showed that individuals with antipsychotic dispensations during follow-up had no increased risk (473 events pre-initiation vs. 444 post-initiation; RR 0.94, 95% CI 0.82–1.07). Among individuals without antipsychotic dispensations, the number of events was small and increased non-significantly from 20 pre-initiation to 26 post-initiation (RR 1.30, 95% CI 0.73–2.33; see Fig. 4).

**Fig. 5:**
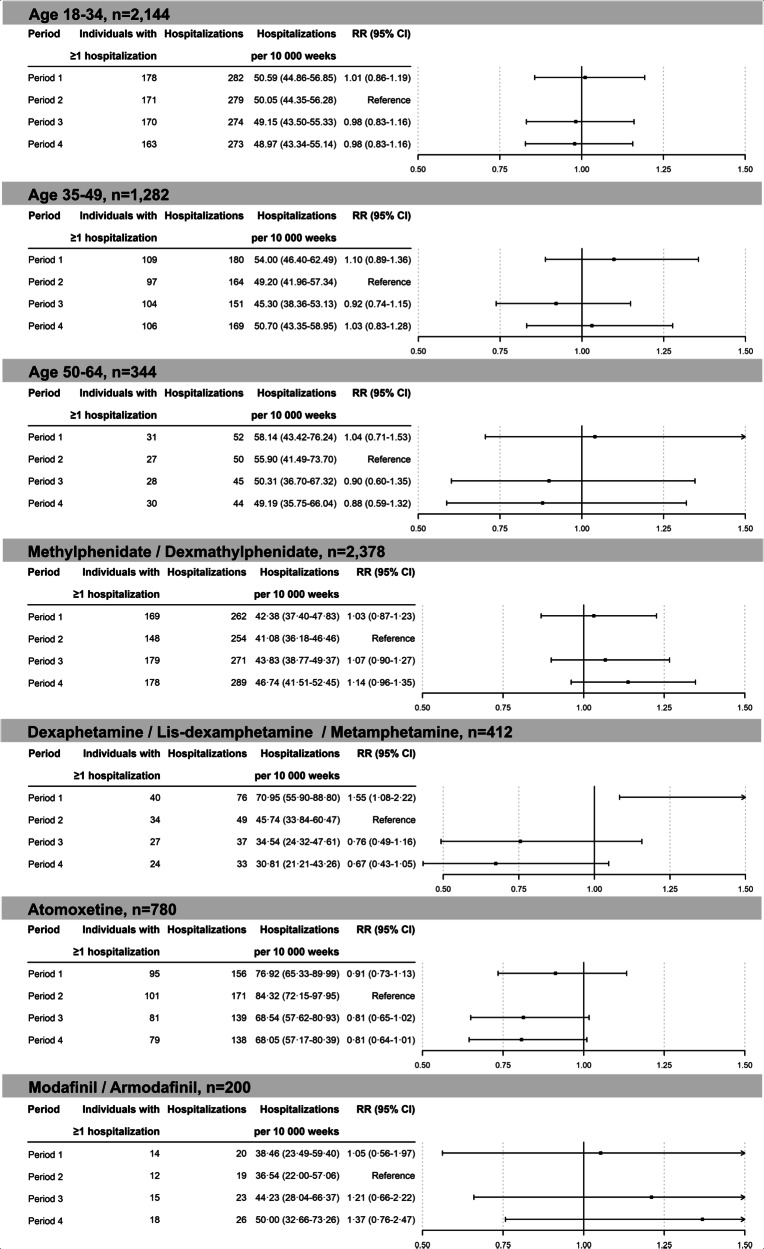
Depiction of the rate ratio of psychotic events before (period 1) and after initiation (period 3 and 4) of psychostimulant treatment (reference: period 2) for different age groups, including age 18–34 years (N = 2144), 35–49 years (N = 1282) and 50–64 years (N = 344) and for different medications, including (dex-)methylphenidate (N = 2378), (lis-)dexamphetamine & methamphetamine (N = 412), atomoxetine (N = 780) and modafinil & armodafinil (N = 200). (Period 1: the 6-month period starting one calendar year before treatment initiation. Period 2: the 6-month period before treatment initiation. Period 3: the 6-month period after treatment initiation. Period 4: the 6-month period staring 6 months after treatment initiation; CI = Confidence Interval, RR=Rate Ratio).

Subgroup analyses by medication type found no significant change in psychosis risk with any specific medication class. Among individuals initiating amphetamine-type psychostimulants, the number of psychotic events decreased from 49 in the pre-initiation period to 37 post-initiation (P3 versus P2: RR 0.76, 95% CI 0.49–1.16). Similarly, for atomoxetine, events decreased from 171 to 139 (RR 0.81, 95% CI 0.65–1.02). In the methylphenidate/dexmethylphenidate group, events increased slightly from 254 to 271 (RR 1.07, 95% CI 0.90–1.27), and for modafinil/armodafinil, events rose from 19 to 23 (RR 1.21, 95% CI 0.66–2.22; see Fig. 5). Confidence intervals across all groups substantially overlapped, indicating no clear evidence of differential risk between medication classes.

Age-group analyses similarly showed no significant risk increase across any category (P3 versus P2: 18–34 years: RR 0.98; 35–49 years: RR 0.92; 50–64 years: RR 0.90; see Fig. 5).

When we restricted the outcome to substance-induced psychosis specifically (ICD-10 code F1x.5), findings remained consistent with the primary analysis (P3 versus P2: RR 0.86, 95% CI 0.65–1.14; see Supplementary Table S1).

Finally, to examine whether findings held in less severe psychotic disorders, we repeated the analysis after excluding individuals with a history of schizophrenia-spectrum and organic psychotic syndromes (N = 2620 individuals excluded). Results were comparable to the full-cohort analysis (see Supplementary Figure S2), suggesting that the absence of increased psychosis risk generalizes across different psychotic disorder diagnostic categories.

## Discussion

The presented work examined the risk of psychosis in 3,770 individuals with history of psychosis after initiation of psychostimulant treatment. Expanding the findings of previous work in adolescents that mainly focused on (dex-)methylphenidate [13, 14], we found no increase in psychotic events after initiation of psychostimulant or atomoxetine treatment among adults with history of psychosis. Specifically, we found no evidence of an increase in the risk of psychotic events when comparing the two 6-month periods one year before treatment initiation to the two 6-month periods one year after. Contrarily, during the six months after treatment initiation we observed an 8% reduction, although non-significant, in the risk of psychosis. These findings challenge the concerns that psychostimulant treatment might increase the risk of psychotic events among individuals with a history of psychosis.

Secondary analyses of medium-term risks also found no increase in the risk of psychotic events among individuals with history of psychosis. Additional sensitivity analyses with shorter eight-week observation periods before and after treatment initiation confirmed these findings, arguing against a transient short-term risk increase.

From a clinical perspective, these findings suggest that, in adults with a history of psychosis initiation of psychostimulants or atomoxetine is not associated with a short- or medium-term increase in hospitalization for psychosis. This challenges the notion that such medications are categorically contraindicated in all patients with prior psychosis and supports guideline recommendations that allow for their cautious use in selected individuals. For clinicians these results are reassuring, when considering psychostimulants or atomoxetine for patients with clinically significant attentional or cognitive symptoms, provided that psychotic symptoms are adequately stabilized and that careful monitoring for emerging or worsening psychotic symptoms is implemented during and after treatment initiation.

Regarding the outcome definition, we used a broad primary outcome of any hospitalization for psychosis, rather than restricting to substance-induced psychosis (ICD-10 F1x.5), for the following reasons: in clinical practice, when stimulant-treated patients with a known psychotic disorder experience symptom exacerbation, clinicians often code the episode under the primary psychotic diagnosis (e.g., schizophrenia) rather than as substance-induced psychosis. Thus, relying solely on F1x.5 diagnoses would substantially underestimate the true frequency of stimulant-associated clinical deterioration. However, substance-induced psychosis (F1x.5) is conceptually most relevant to the hypothesis of direct drug-induced psychotic symptoms, as ICD-10 F1x.5 requires that onset of psychotic symptoms are judged by the clinician to be directly attributable to the substance. Accordingly, in sensitivity analyses we restricted outcomes to F1x.5 diagnoses, which produced findings consistent with the primary analyses. The convergence of results across these two outcome definitions strengthens the inference that psychostimulant or atomoxetine initiation was not associated with an increase in either broadly defined psychotic deterioration or substance-attributed psychotic events.

The sensitivity analyses in both sexes, different age groups, individuals with and without concurrent prescription of antipsychotics and different medication groups separately confirmed the finding that the risks of psychotic events did not increase significantly after treatment initiation. What stood out was a descriptively higher risk in individuals that did not receive antipsychotic prescriptions during follow-up.

The point estimate indicated that there was a non-significant 30% increase in the risk for psychotic events from before to after treatment initiation in the group without antipsychotic treatment, while a non-significant 6% decrease in the risk for psychotic events were observed in individuals that received antipsychotic treatment. This finding is in line with previous research [14] and indicates that psychosis risk might be lower when psychostimulant treatment is combined with antipsychotics. This pattern is clinically consistent with the hypothesis that adequate antipsychotic coverage may mitigate the dopamine-releasing effects of psychostimulants, thereby protecting against symptom exacerbation. It should be noted that these changes in risks were non-significant and that confidence intervals of both groups overlapped. Thus, the presented data cannot definitely answer whether risk in individuals with and without concurrent antipsychotic treatment differ. The lack of statistical significance likely reflects limited power rather than a definitive absence of interaction. Future studies with larger samples are needed to definitively test whether antipsychotics modify the safety profile of stimulants in adults with a history of psychosis.

The medication subgroups showed numerically divergent patterns. For amphetamine-type stimulants (RR 0.76) and atomoxetine (RR 0.81) point estimates indicated a decrease in risk, while for methylphenidate point estimates (RR 1.07) indicated a slight increase, though these differences were not statistically significant. Overlapping confidence intervals across medication classes and small event counts in some subgroups preclude definitive conclusions about differential medication effects. In addition, results are partially in contrast to previous studies, which reported an increased risk of psychosis (incidence) during treatment with amphetamine-like psychostimulants [11, 18, 19]. Still, other studies also found no evidence that the risks of psychotic events were greater in those receiving amphetamine-like psychostimulants could not but in line with other research [14]. Different findings might be explained by different observational periods and age groups. Presented work expands previous findings on adolescent samples by investigating effects in adult populations.

### Strengths and limitations

The strengths of the presented study include the large number of participants and the naturalistic setting, in which we used register-data with nation-wide coverage. The study provides a longer observation period than most clinical trials and includes a large population of individuals with a history of psychosis. Moreover, the use of a within-individual design adjusts for between-individual differences, which would not be possible to adjust for in a conventional epidemiological approach. This design provides an improved strategy for handling selection effects and confounding by indication in pharmaco-epidemiological studies.

A limitation of the study is that, although the design allows for adjustment of otherwise unmeasured confounders, it does not adjust for changes that might have occurred during the 24-month observation period. Further limitations of our study include missing information on dosage, missed doses, and the possibility that psychotic events not resulting in hospital visits might have been missed. To account for potential risk of bias by concomitant treatment with antipsychotics, we repeated the primary analysis among individuals that did not receive a prescription for antipsychotics during the one-year follow-up. Results corroborated the findings of the primary analysis. The statistical model required that individuals that died during the observational period needed to be excluded. These might include severe patient cases. Hence, results might not be generalizable to these severe cases.

The cohort included individuals with a range of psychotic disorders, including schizophrenia-spectrum disorders (F2x) and affective psychoses (F3x). While the evaluation of the question whether exacerbations are more likely in schizophrenia-spectrum disorders than in affective psychoses is certainly of clinical interest, our within-individual design and the number of events did not allow for subgroup analyses. Hence, future studies should address this question in larger datasets or pooled analyses

To address potential diagnostic heterogeneity in the main cohort, we conducted a sensitivity analysis restricting to individuals without a history of schizophrenia-spectrum or organic psychotic disorders. Results from this analysis were consistent with the primary findings, suggesting that the conclusion of no increased psychosis risk generalizes across different diagnostic subgroups within the population with a history of psychosis.

We included individuals who did not receive psychostimulant or atomoxetine prescriptions 1.5 years prior to the index dispensation. Even though our selection was based on empirical data, it could be that individuals with longer medication-free intervals may represent those with good tolerability and lower inherent psychosis risk.

To address this, we conducted additional sensitivity analyses restricting the cohort to individuals with no recorded psychostimulant or atomoxetine dispensations throughout the entire available look‑back period, extending up to 16 years prior to the index dispensation (i.e., the maximum time span covered by the registry data; N = 3394). Results from this more restrictive definition were descriptively similar to those of the main analysis, with point estimates of 0.94–0.96 for the primary comparison (period 2 vs. period 3) and confidence intervals overlapping unity (see supplementary Table S2). The consistency of results across different washout periods supports the robustness of presented findings.

A total of 50 individuals (1.31%) died during the observational period and were excluded, with notable proportions due to suicide (n = 16) and accidents (n = 16). It should be noted that the necessity to exclude these individuals from within-individual analyses may limit the generalizability of presented results to very severe patient cases. Still, the small proportion of excluded individuals suggests minimal risk of bias for the main estimates.

## Conclusion

In conclusion, contrary to clinical concerns, our study did not detect an increased risk of psychosis after starting psychostimulant treatment among individuals with a history of psychotic events.

## Supplementary information

Supplements to Prescription psychostimulants, atomoxetine and the risk of psychosis in adults with history of psychosis: a population-based cohort study

## Data Availability

No individual de-identified participant data will be shared. The data used in this study are derived from Swedish national registries, which are subject to strict confidentiality and data protection regulations. Access to these data is restricted by Swedish law and is granted only for specific research purposes following ethical approval and a data request process. No additional related documents, such as the study protocol or statistical analysis plan, will be made available beyond what is published in the article. Researchers interested in accessing similar data must apply directly to the relevant Swedish authorities, such as the National Board of Health and Welfare (Socialstyrelsen) and Statistics Sweden (SCB), following their established procedures. Access is granted on a case-by-case basis and requires an approved ethical review.
