# Supplementary material for: Prescription psychostimulants, atomoxetine and the risk of psychosis in adults with history of psychosis: a population-based cohort study
Source: Transl Psychiatry. 2026 Mar 31;16:226. doi: 10.1038/s41398-026-03998-4 (PMC13043684; doi:10.1038/s41398-026-03998-4)
Supplement: Supplementary file 1 — Supplements to Prescription psychostimulants, atomoxetine and the risk of psychosis in adults with history of psychosis: a population-based cohort study [file 41398_2026_3998_MOESM1_ESM.docx]

**Supplements to**

*Prescription psychostimulants, atomoxetine and the risk of psychosis in adults with history of psychosis: a population-based cohort study*

**Content:**

- **Supplementary Tables**
  - **Supplementary Table S1.** Rate Ratio of substance-induced psychotic events (main diagnosis F1x.5) for individuals with history of psychosis (N=3 770) before (period 1) and after initiation of treatment with psychostimulants or atomoxetine (periods 3 and 4), compared to period 2 (reference).
  - **Supplementary Table S2.** Rate Ratio of psychotic events before (period 1) and after initiation of treatment with psychostimulants or atomoxetine (periods 3 and 4), compared to period 2 (reference) among individuals with history of psychosis who had no recorded prescriptions for psychostimulants or atomoxetine during the entire available look‑back period, up to 16 years prior to the index dispensation (i.e., the maximum duration covered by the registry data) (N = 3,394).
- **Supplementary Figures**
  - **Supplementary Figure S1.** Depiction of the Rate Ratio of psychotic events for individuals with history of psychosis (N=3 770) during the 8-week period after initiation of treatment with psychostimulants or atomoxetine, compared to the 8-week period directly before treatment initiation.
  - **Supplementary Figure S2.** Rate Ratio of hospitalization due to psychosis before (period 1) and after initiation of treatment with psychostimulants or atomoxetine (periods 3 and 4), compared to period 2 (reference) among individuals with history of psychosis, after excluding individuals with history of history of schizophrenia (F20.x), persistent delusional disorders (F22.x), schizoaffective disorders (F25.x), organic hallucinosis (F06.0), and organic delusional disorders (F06.2) (n=2 620).

## Supplementary Table S1. Rate Ratio of substance-induced psychotic events (main diagnosis F1x.5) for individuals with history of psychosis (N=3 770) before (period 1) and after initiation of treatment with psychostimulants or atomoxetine (periods 3 and 4), compared to period 2 (reference). Period 1: the 6-month period starting 1 calendar year before treatment initiation. Period 2: the 6-month period before treatment initiation. Period 3: the 6-month period after treatment initiation. Period 4: the 6-month period starting 6 months after treatment initiation. RR = Rate Ratio.

| **Period** | **Participants with at least one hospitalization** | **Number of hospitalizations** | **RR (95% CI)** |
| --- | --- | --- | --- |
| 1 | 73 | 104 | 1.13 (0.85-1.49) |
| 2 | 63 | 92 | 1 (reference) |
| 3 | 58 | 80 | 0.86 (0.65–1.14) |
| 4 | 50 | 65 | 0.71 (0.51–0.97) |

## Supplementary Table S2. Rate Ratio of psychotic events before (period 1) and after initiation of treatment with psychostimulants or atomoxetine (periods 3 and 4), compared to period 2 (reference) among individuals with history of psychosis who had no recorded prescriptions for psychostimulants or atomoxetine during the entire available look‑back period, up to 16 years prior to the index dispensation (i.e., the maximum duration covered by the registry data) (N = 3,394). Period 1: the 6-month period starting 1 calendar year before treatment initiation. Period 2: the 6-month period before treatment initiation. Period 3: the 6-month period after treatment initiation. Period 4: the 6-month period starting 6 months after treatment initiation. RR = Rate Ratio.

| **Individuals without psychostimulants or atomoxetine in up to 16 years prior to observational period** | | | |
| --- | --- | --- | --- |
| **Period** | **Participants with at least one hospitalization** | **Number of hospitalizations** | **RR (95% CI)** |
| 1 | 278 | 450 | 1.04 (0.91-1.19) |
| 2 | 260 | 432 | 1 (reference) |
| 3 | 263 | 410 | 0.95 (0.83–1.09) |
| 4 | 264 | 413 | 0.96 (0.84–1.09) |

## Supplementary Figure S1. Depiction of the Rate Ratio of psychotic events for individuals with history of psychosis (N=3 770) during the 8-week period after initiation of treatment with psychostimulants or atomoxetine, compared to the 8-week period directly before treatment initiation. Period 2: the 8-week period before treatment initiation. Period 3: the 8-week period after treatment initiation. RR=Rate Ratio.


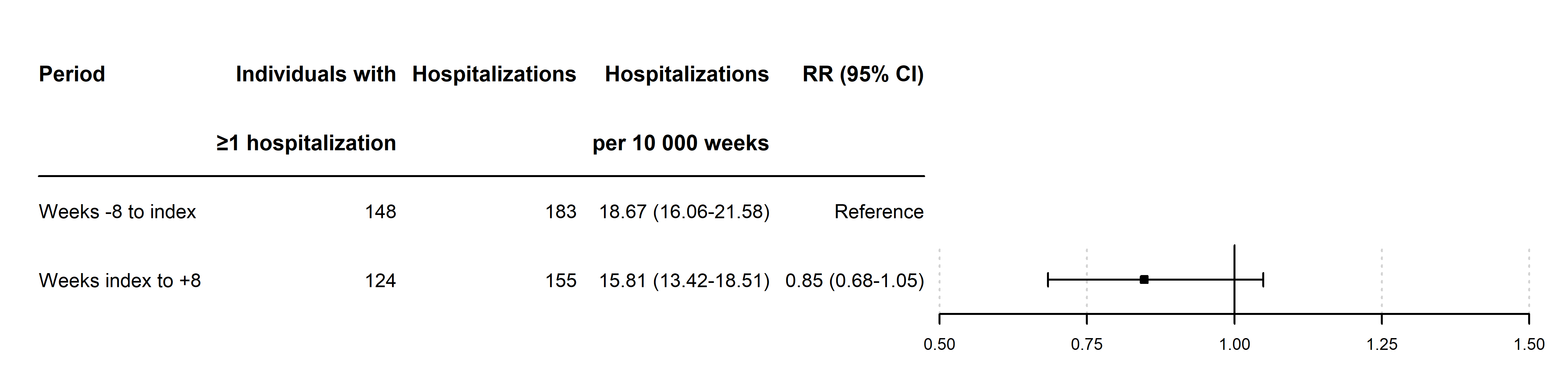


## Supplementary Figure S2. Rate Ratio of hospitalization due to psychosis before (period 1) and after initiation of treatment with psychostimulants or atomoxetine (periods 3 and 4), compared to period 2 (reference) among individuals with history of psychosis, after excluding individuals with history of history of schizophrenia (F20.x), persistent delusional disorders (F22.x), schizoaffective disorders (F25.x), organic hallucinosis (F06.0), and organic delusional disorders (F06.2) (n=2 620). Period 1: the 6-month period starting 1 calendar year before treatment initiation. Period 2: the 6-month period before treatment initiation. Period 3: the 6-month period after treatment initiation. Period 4: the 6-month period starting 6 months after treatment initiation. RR = Rate Ratio.

**
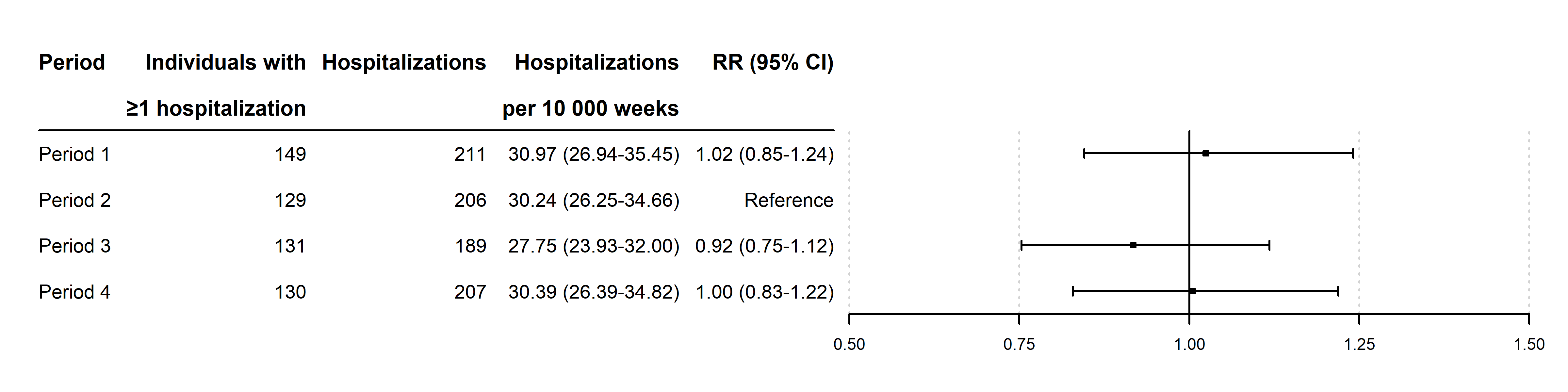
**
